# Recommendation for changes to the guidelines of trauma patients with potential spinal injury within a regional UK ambulance trust

**DOI:** 10.29045/14784726.2022.12.7.3.59

**Published:** 2022-12-01

**Authors:** Alan Cowley, Magnus Nelson, Claire Hall, Simon Goodwin, Dhushyanthan Surendra Kumar, Fionna Moore

**Affiliations:** South East Coast Ambulance Service NHS Foundation Trust ORCID iD: https://orcid.org/0000-0002-3093-4395; South East Coast Ambulance Service NHS Foundation Trust; South East Coast Ambulance Service NHS Foundation Trust; South East Coast Ambulance Service NHS Foundation Trust; University Hospitals Coventry & Warwickshire; South East Coast Ambulance Service NHS Foundation Trust

**Keywords:** pre-hospital, spinal injuries, trauma

## Abstract

**Background:**

Spinal assessment and immobilisation has been a topic of debate for many years where, despite an emerging evidence base and the delivery of new guidance overseas, little has changed within UK pre-hospital practice. Since 2018, South East Coast Ambulance Service NHS Foundation Trust has spent time working with local trauma networks and expertise from within the region and international colleagues to develop a set of C-spine assessment and immobilisation guidelines that reflect the current best available international evidence and significant changes in international pre-hospital practice from settings such as Scandinavia and Australasia.

**Methods:**

A specialist group was commissioned to review the topic of pre-hospital spinal immobilisation and explore potential for evidence-based improvement. In conjunction with local trauma networks, subject matter experts and a thorough review of recent literature, a series of recommendations were made in order to improve spinal care within the authoring trust.

**Results:**

Seven recommendations were made, and an updated set of guidelines produced. These included the removal of semi-rigid collars from pre-hospital spinal immobilisation; the creation of two tiers of patients to ensure that the high-risk and low-risk populations are considered separately and an accompanying decision tool to safeguard both cohorts; an increased emphasis on the risk of spinal injury in the frail and older patient; an emphasis on spinal motion restriction rather than rigid immobilisation; an increased emphasis on self-extrication; and the use of a marker for emergency departments.

**Summary:**

An updated set of guidance has been produced using a combination of specialist and expert opinion alongside a literature review with close involvement of key stakeholders, both public and professional. The new guidance helps to ensure a patient-centred approach where each person is considered an individual with their risk of injury and management measures tailored to their specific needs.

## Background

Traumatic injury is common, with acute injury to the spinal column being relatively rare and spinal cord injury rarer still, occurring in only 0.5–3.0% of blunt trauma incidents (Cameron et al., 2005; Kumar et al., 2018), with this number dropping even lower in penetrating trauma (Garcia et al., 2014). Spinal care within UK ambulance trusts is based on historical practice and supported only by low-quality evidence. It directs that where spinal injury may have occurred a strict regime of manual immobilisation should be adopted immediately, followed by a triple-point approach involving a supine patient, semi-rigid collar and head blocks. In recent years, and since the last updated UK ambulance and faculty of pre-hospital care guidance (Joint Royal Colleges Ambulance Liaison Committee (JRCALC)) (Brown et al., 2016), there has been a shifting balance of opinion, and evidence, that this may often not be the best approach and that an individual patient-centred approach may be more beneficial. Adoption of alternative approaches aligned with changing evidence-based practice in our region’s major trauma centres (MTCs) and further afield, such as the ‘soft collar’ approach in Queensland (Asha et al., 2021), has provoked debate within our trust as to whether there was scope to review and potentially change our approach to immobilisation.

With the evolution of the major trauma system in England, there has been an increasing recognition of the volume of trauma in frail and elderly patients, sometimes referred to in the United Kingdom as ‘silver trauma’ (Alshibani et al., 2020). Local and Network incident reporting has shown that the propensity for occult spinal injury is higher in this group but has not always been at the forefront of pre-hospital clinicians’ minds and current standard packaging may not take account of the challenges of immobilisation in this patient cohort (Atinga et al., 2018).

South East Coast Ambulance Service NHS Foundation Trust (SECAmb) is an urban, suburban and rural NHS-funded ambulance service that broadly encompasses the counties of Sussex, Surrey, Kent and North East Hampshire and receives nearly 862,000 calls each year.

In June 2018, commissioned by JRCALC, a working group was established within SECAmb to review the topic of pre-hospital spinal immobilisation and explore potential for evidence-based improvement. The group consisted of the trust’s medical director and assistant medical director (both consultants in emergency medicine, and with a large amount of pre-hospital experience, primarily in the air ambulance setting), a selection of CCPs and a clinical education specialist paramedic. The brief of the group was to identify the potential for change in the trust’s guidance and practice. The internal group was augmented with the addition of external clinical expert opinions from a variety of backgrounds and organisations. The group elected to tackle the problem by reviewing and assessing two questions:

- Who should be immobilised?
  - Is there a decision tool that can address the problem of under immobilisation in the high-risk population, without over-immobilising the low-risk patients?
- How to immobilise?
  - Is the current guidance of triple-point immobilisation in the vast majority of patients the most appropriate and evidence-based method?

## Methods

The group adopted a varied approach to assessing and addressing the issues:

- Literature search – two of the group independently performed literature searches, utilising PubMed and CINAHL. Only articles published since 2010 (up until early 2018) were considered in order to identify any new evidence since the most recent sets of published UK guidelines. Strict MESH terms were not used due to the scope of the project.
- Trauma network liaison – the group actively sought the views of three regional major trauma networks within our area of operation in order to better understand how the emergency department and pre-hospital sector could work together to improve patient experience.
- External liaison – the group actively liaised with subject matter experts outside of the trust and local networks. These included ambulance services in different countries and specialists in fields outside of the pre-hospital and emergency sectors, where change had already occurred. Neurosurgeons, tissue viability practitioners, intensive care unit clinicians and rescue services were consulted.
- Specialist opinion – due to the makeup of the group, it was considered a specialist group. Experience within the group included many years of pre-hospital specialist working, including several members with current and previous helicopter emergency medical services experience in a variety of trusts.

### Patient and public involvement

Direct patient feedback and experience was one of the driving factors behind this work, the lessons learned from previous serious incidents being crucial to the inception of the project. In the latter stages of the work, key stakeholders that included patient groups were asked to comment on the guidance and positive feedback was received. The aim of this was to ensure that the direction, intent and rationale of the guidance were in keeping with stakeholder expectations. Further work will be published in due course that takes account of the patient experience and feedback now that the guidance has been rolled out.

The involvement of the public stakeholders helped to reaffirm the panel’s stance that the improvement project is ethically sound. This position is based on the following principles:

- The improvement plan is focused on ensuring a more bespoke approach to each patient.
- The recommendations will focus on changing decision making and intervention to mitigate the risk of evidenced harm.
- The recommendations will utilise currently available and validated equipment and decision tools.

## Results

The group met bi-monthly across the 18-month period of development to review the academic material and work through the initial questions posed. This allowed the group to make seven recommendations (two relating to ‘Who should be immobilised?’ and five relating to ‘How to immobilise?’) for change to the current UK ambulance service guidelines, which were supported by the local networks and by the formal processes of the trust as being appropriate for the care of patients within our service. The outcomes of the process allowed the introduction of the recommendations to the service (Supplementary 1) and an understanding that as part of the introduction there would be a two-year review of implementation and outcome. These recommendations are detailed below.

### Recommendation 1: introduction of a new cervical clearance tool that discriminates between low- and high-risk patients

#### Rationale and evidence base

**Figure 1:**
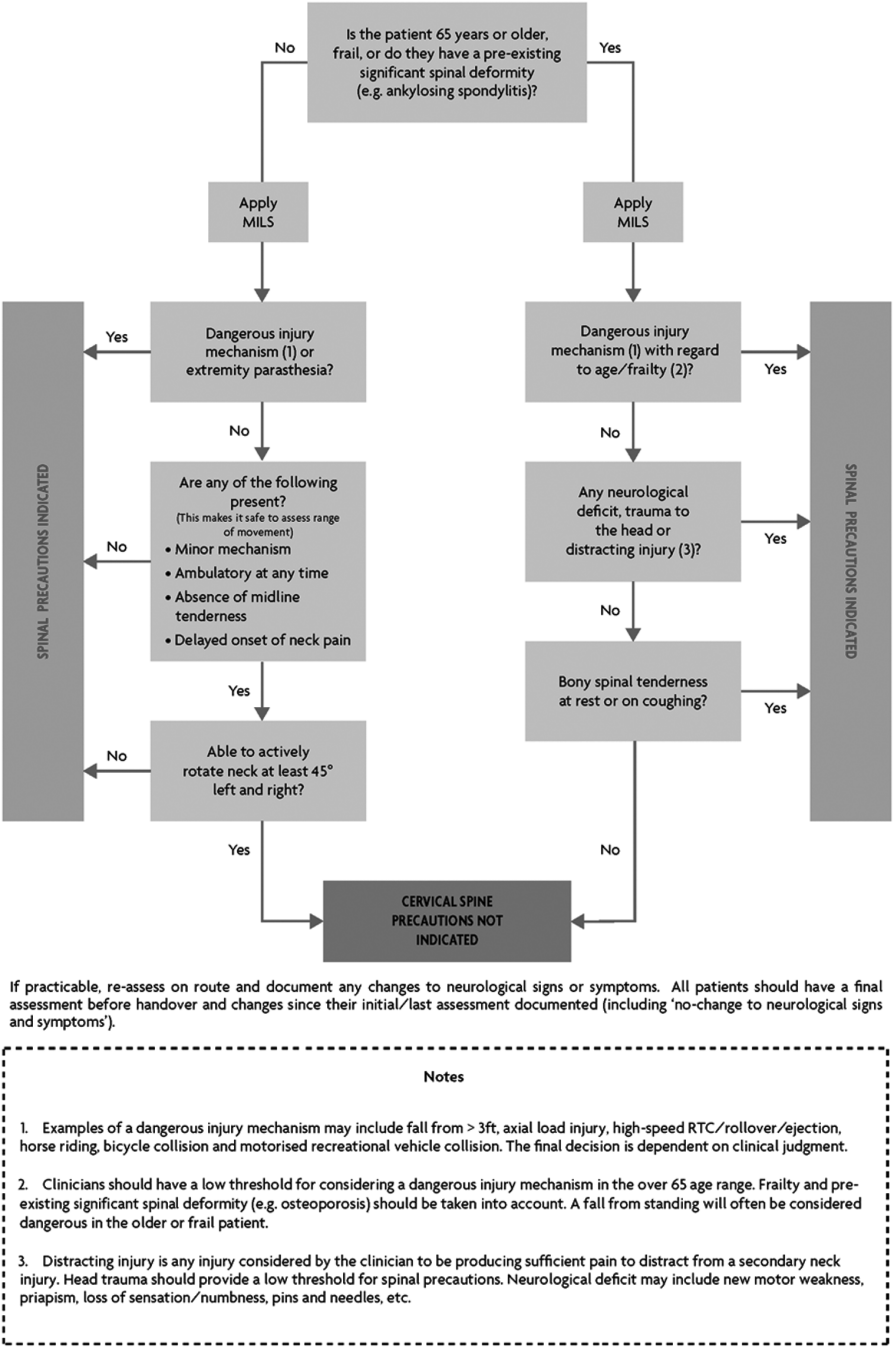
Figure 1. Spinal precautions decision tool.

The previous spinal decision tool was an unvalidated merger of the Canadian C-spine and NEXUS tools (Connor et al., 2013). The group felt that validated tools would provide a far more robust method of deciding when cervical spine immobilisation was not indicated. Due to recommendations 2 and 3, it was felt that the tool should reflect the differing patient populations when it comes to trauma. Serious spinal injury in the young, healthy population is very rare in low mechanism incidents, but this is not the case in the older/frail population (Herron et al., 2017; London Major Trauma System, 2017). The Canadian C-spine rules are a validated and internationally recognised decision tool which also has pre-hospital validation (Vaillancourt et al., 2009). However, it mandates imaging in all patients over 65 years of age and while this is not the same as mandating immobilisation, it remains a prudent approach to minimise movement in any spine which requires a bony injury to be ruled out radiographically. This equates to a significant proportion of the population who may not have required conveyance to hospital but as a result of this tool require not only conveyance but imaging and associated work-up. NEXUS provides a far more pragmatic approach in these patients, and with evidence suggesting its effectiveness can be improved in this patient population (Tran et al., 2016), it was seen as a validated and risk-averse decision tool in this particular cohort of patients. Its wording has been adjusted by the group to make it more relevant to UK pre-hospital practice, but its principles remain (see Figure 1).

In the under-65 population, the Canadian C-spine rule would empower the clinician to safely assess the range of neck motion in those patients who have not experienced a dangerous mechanism, while maintaining a robust and validated decision tool. Over-immobilisation and over-triage of these patients is of significant concern to the ambulance service and trauma networks due to the high volume of low-mechanism incidents in this low-risk group.

In the over-65 population, the modified NEXUS rule will account for the increased susceptibility of this patient group at lower mechanisms, while still allowing the clinician to safely eliminate the need for imaging (and, therefore, assumed need for immobilisation) while using a validated tool.

It should be noted that it is impossible to account for all dangerous and minor mechanisms in a list, and a degree of subjectivity will always exist. In addition, the term ‘comfortable in a sitting position’ has been removed from the Canadian C-spine rules, as a significant proportion of road traffic collision patients will be encountered sitting in their car, regardless of injuries. This omission is likely to serve only to make the tool more risk averse, and not impact on its sensitivity.

Finally, there is no validated spinal clearance tool for the paediatric population, despite the relatively lower risk. While there is no evidence to support or refute the use of this tool in paediatrics, we deem it suitable to be applied where the patient is able to co-operate and deemed competent to assess using the listed criteria. Where this is not possible (generally in the very young), then a risk versus benefit assessment should be made and a low threshold for a ‘position of comfort’ approach (as per recommendation 4) until specialist assessment can be sought. This is already supported in the United Kingdom by NICE and ATLS (advanced paediatric life support) teaching (Copley et al., 2019).

### Recommendation 2: increased emphasis on risk of spinal injury in high-risk selected patient populations

#### Rationale and evidence base

Elderly and frail trauma is an increasingly important and documented issue in acute healthcare (Herron et al., 2017; London Major Trauma System, 2017; Trauma Audit and Research Network, 2017). A fall from standing in an older, frail patient is now the highest group within the Trauma Audit and Research Network presenting with major trauma. Within the authoring trust, there have been several serious incident reviews regarding ‘missed’ cervical spine injuries in this patient population. Root cause analysis has suggested this is down to a lack of appreciation for the increased risk of spinal injury, despite an apparently minor mechanism (Trauma Audit and Research Network, 2017). While this has been known for a significant period of time, it warrants an emphasis within the guidelines in an attempt to reduce the number of missed injuries. A modified NEXUS approach to this patient group has recently been suggested (Tran et al., 2016), which includes ‘head and facial trauma’ within the distracting injury section, and this has been incorporated.

Manual in-line stabilisation must remain an absolute priority in the high-risk patient with a loss of protective muscle tone (i.e. unconscious) and with a high-risk mechanism. This is because the protective measures present in a conscious patient (e.g. increased muscle tone around the injury site, self-splinting of injury due to pain) are likely absent, or reduced, in this patient group.

### Recommendation 3: removal of semi-rigid collars from use

#### Rationale and evidence base

The use of semi-rigid collars is an increasingly controversial topic in pre-hospital care and many advanced trauma teams and emergency departments now do not employ them as their primary standard of care. Current UK ambulance guidelines (Brown et al., 2016) suggest they should be omitted only if counterproductive or contra-indicated.

#### Efficacy and possible harm

There is a lack of any evidence, outside of expert opinion and established practice, to show the true efficacy of semi-rigid cervical collars (Horodyski et al., 2011; Oteir et al., 2015). The group acknowledge that absence of evidence is not evidence of no benefit, but studies and expert consensus continue to emerge questioning their ability to restrict spinal motion (Kornhall et al., 2017), and providing evidence that the movement that is allowed is too great to show true efficacy. Increasingly, advanced trauma teams and emergency departments are dispensing with it as an adjunct to cervical immobilisation. In recent years, there has also been an emerging evidence base to suggest the detrimental effects of semi-rigid collars, particularly in older and frail patients, and the association with pressure sores (Ham et al., 2016; Worsley et al., 2018). They have also been shown to restrict mouth opening, increase intra-cranial pressure (Maissan et al., 2018), increase pain (Ham et al., 2016) and agitation, cause mandibular nerve palsy, increase motion in the high cervical spine, encourage a false sense of full immobilisation in the rescuer and worsen neurological outcomes in patients with pre-existing spinal conditions (Holla, 2012; Kornhall et al., 2017; Sundstrom et al., 2014). Studies on cadavers have suggested that injury could be aggravated depending on the fracture site (Ben-Galim et al., 2010). Spinal motion capture studies using healthy volunteers or cadavers have been used to accurately measure immobilisation techniques. Perry et al. (1999) identified that various techniques and equipment that are routinely used in the traditional method of immobilisation do not eliminate neck motion during extrication or transport. Other studies have identified that using conventional techniques and equipment may actually increase neck motion (Dixon et al., 2014; Engsberg et al., 2013). Healthy volunteers have also reported pain and discomfort while being immobilised.

#### Semi-rigid collars should no longer be used in pre-hospital spinal immobilisation

Taking into account the increasing evidence base of harm, and the lack of proven efficacy, the semi-rigid collar should no longer be used in pre-hospital spinal care. The group felt the risk of collars significantly outweighed any potential benefit.

### Recommendation 4: increased emphasis on ‘spinal motion restriction’ and creation of a two-tier system for immobilisation

#### Rationale and evidence base

In consultation with advanced trauma teams and the major trauma networks, it was felt that immobilisation on a split-extrication device (e.g. orthopaedic scoop stretcher) was the optimum method until handover at the emergency department. This ensured maintenance of a minimal handling approach – an important concept, as many of these patients may have multi-system trauma. However, the group acknowledge that this may be sub-optimal in a significant population of patients who are at highest risk from hard-surface stretcher systems such as a scoop, and from a prolonged restricted fully supine position (e.g. older, bariatric, paediatric patients, agitated patients and those patients with spinal abnormalities) (Abram & Bulstrode, 2010).

For this cohort in particular, an emerging body of opinion is suggesting that spinal motion restriction, in contrast to rigid immobilisation, is a safe and effective method of protecting any injury until imaging can take place (Swartz et al., 2018). This builds on the concept of self-immobilisation first suggested by Hauswald (2013) and built upon in several small-scale studies (Hunt et al., 2016; Tatum et al., 2017). There is no evidence to show that spinal motion restriction is superior to rigid immobilisation (Swartz et al., 2018) but given the degree of risk of harm in this patient group, it is recommended that this group of patients has spinal motion restriction employed by a way of a ‘position of comfort’ approach. This is likely to be within a supportive carrying device but does not exclude any position that promotes spinal motion restriction in that patient. It was felt that this group of patients presents less commonly with high mechanism polytrauma (London Major Trauma System, 2017; Trauma Audit and Research Network, 2017), and so the benefits of staying on the scoop are outweighed by its risks.

### Recommendation 5: use of scoop in low-risk patients and increase of maximum time to 1 hour

#### Rationale and evidence base

As detailed in recommendation 2, there is no strong evidence currently to promote rigid immobilisation above or below spinal motion restriction (Swartz et al., 2018). In consultation with our partner major trauma networks, it was felt that the patients at lowest risk of complications from a restricted supine period on a hard surface should be ‘packaged’ on a scoop with head blocks and tape. The scoop should be secured for conveyance according to local policy; this will often be inside a vacuum mattress. The importance of minimal handling in the high mechanism blunt trauma patient is well documented (Moss et al., 2013), and this is most easily achieved using a scoop for transit. It also aids in reducing on-scene times, which has been shown to have an effect on mortality in the trauma patient (Pham et al., 2017). No evidence exists to demarcate the time frame for hard surface transit in relation to the risk of pressure sore; older studies focus on timescales of several hours which are outside that of the standard pre-hospital environment in the United Kingdom (Kornhall et al., 2017). They do suggest the need for an upper limit, however. This was chosen as 1 hour, which coincides with the agreed isochrone for direct conveyance to our regional MTC within our major trauma decision tree. Outside of this time frame, a risk–benefit analysis should be adopted and consideration given to packaging the patient without a scoop.

### Recommendation 6: use of a ‘marker’ for emergency departments

#### Rationale and evidence base

The shift to a ‘position of comfort’ approach in a large cohort of patients presents a potential risk that these patients may be overlooked in the emergency department due to their lack of ‘classic’ trauma visual flags (i.e. semi-rigid collar, head blocks, supine position). In consultation with our partner major trauma networks, and networks abroad that have adopted this strategy, it was felt that the application of a ‘marker’ to these patients would reduce the likelihood of a delay in assessment in the emergency department. The type of marker could be decided locally and may take the form of a brightly coloured lanyard or soft collar. In the case of the latter, be aware that there is good evidence to show that these collars do not physically restrict cervical motion, though there is a likelihood that they ‘encourage’ patients to keep their neck still (Connor et al., 2013). However, it should be noted that their use in these guidelines is recommended solely as a marker. No concerns have been documented in terms of difficulty in applying these devices, and certainly they will be no more difficult to apply than a cervical collar, with none of the associated detrimental effects (Rashford & Schultz, 2021). A small study exists showing that older patients spend longer ‘immobilised’ in the emergency department and that softer methods of spinal restriction and methods to speed up their assessment would be beneficial (Edwards et al., 2014). There is not yet any evidence to show whether their application does reduce assessment delays in the emergency department.

### Recommendation 7: increased emphasis on self-extrication and removal of spinal pain as a contra-indication to self-extrication

#### Rationale and evidence base

**Figure 2:**
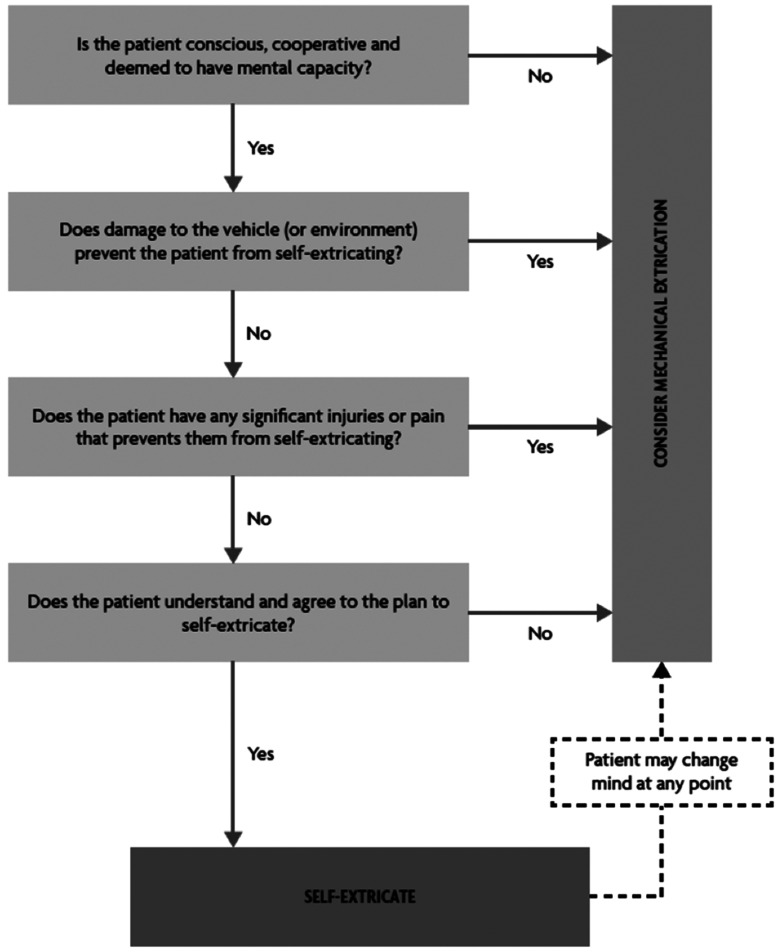
Figure 2. Self-extrication guidance.

There is now much stronger opinion and evidence than previously available regarding the degree of cervical motion that the co-operative patient experiences during self-movement, as opposed to traditional manual extrication techniques (Connor et al., 2013; Cowley, 2014; Cowley et al., 2017; Dixon et al., 2014, 2015). This helps to endorse the view that self-extrication, as well as being quicker, conserves resources, is less emotionally traumatic and is far more effective in the pursuit of spinal motion restriction than traditional extrication. This provides a strong rationale that spinal pain per se should not prevent the encouragement of self-extrication (see Figure 2).

## Dissemination, teaching and monitoring

It is recommended that this change be communicated to the pre-hospital workforce by way of a teaching session or online tool, as it represents a shift in culture for many practising pre-hospital clinicians. A follow-up article will cover the topic of dissemination and teaching for the workforce and provide an update on implementation and any lessons learned.

The new guidance will be monitored closely over the coming years. While it is very difficult to directly measure the primary effects of the guidance, due to the very rare incidence of spinal cord injury, surrogate measures will be used and an implementation study published in the future. Effectiveness of the guidance (as opposed to the implementation) will be monitored using the following:

- direct feedback from trauma networks;
- monitoring of adverse incident reporting; and
- liaison with patient groups.

## Summary

These guidelines are an attempt to standardise our trust’s pre-hospital approach to spinal care and bring it in line with the latest evidence base. They aim to reduce the potential for iatrogenic harm while promoting an individual patient-centred approach, allowing the clinician to move away from a ‘one size fits no-one’ approach. This is particularly important as the concept of ‘silver’ trauma continues to receive a developing focus in pre-hospital and trauma services in line with the recognition that these patients make up a significant part of the major trauma presentations in England. The new guidance will also aim to address the potential problems associated with ‘over triage’ in the low-risk patient and ‘under triage’ in the high-risk patient.

## Acknowledgements

The authors would like to acknowledge Brian Carlin and ASPIRE for their assistance in shaping the direction of this guidance, and Dr. Timothy Nutbeam for his engagement with the work, particularly surrounding self-extrication.

## Author contributions

All authors met the journal definition for authorship. This was not a formal service improvement study. A follow-up study focusing on the implementation will be written in due course. AC acts as the guarantor for this article.

## Conflict of interest

DSK has engaged with the National Institute for Health and Care Research regarding a research proposal for a randomised control trial on semi-rigid collars. Otherwise, there are no competing interests to declare.

## Ethics

Formal ethics approval was not required as the study is not considered research by the NHS Health Research Authority. It was intended as a service development and improvement work.

## Funding

None.
